# A super absorbent polymer containing copper to control *Plenodomus tracheiphilus* the causative agent of mal secco disease of lemon

**DOI:** 10.3389/fmicb.2022.987056

**Published:** 2022-09-08

**Authors:** Soumia El boumlasy, Federico La Spada, Antonella Pane, Antonino Licciardello, Abderrahmane Debdoubi, Nunzio Tuccitto, Santa Olga Cacciola

**Affiliations:** ^1^Laboratory of Materials-Catalysis, Department of Chemistry, Faculty of Science, Université Abdelmalek Essaadi, Tetouan, Morocco; ^2^Department of Agriculture, Food and Environment, University of Catania, Catania, Italy; ^3^Consorzio per lo Sviluppo dei Sistemi a Grande Interfase, Catania, Italy; ^4^Department of Chemical Sciences, University of Catania, Catania, Italy

**Keywords:** copper, antifungal activity, MIC, MFC, eco-friendly products, new technology, AAS, ToF-SIMS

## Abstract

The aim of this study was to determine the effectiveness of a Super absorbent polymer (SAP) containing copper (SAP-Cu) in controlling mal secco disease (MSD) of lemon caused by the fungus *Plenodomus tracheiphilus*. Super absorbent polymer containing copper was characterized by atomic absorption spectrometry (AAS) and UV-VIS spectroscopy. *In vitro* tests were performed to determine the inhibitory effects of SAP-Cu against the pathogen on both potato-dextrose-agar medium and naturally infected lemon cuttings. Super absorbent polymer was able to absorb up to about 200 and 30 times its weight of ionized water and copper (II) sulfate solution (Cu^2+^ ions at the concentration 236 mM), respectively. The distribution of copper released on twigs after 24 h of contact with SAP-Cu was determined by secondary ion mass spectrometry with time-of-flight analyzer (ToF-SIMS). Super absorbent polymer containing copper significantly inhibited the viability of *P. tracheiphilus* in lemon twigs. Overall, the results of this study showed that the SAP could be a suitable carrier of antifungal compounds.

## Introduction

**FIGURE 1:**
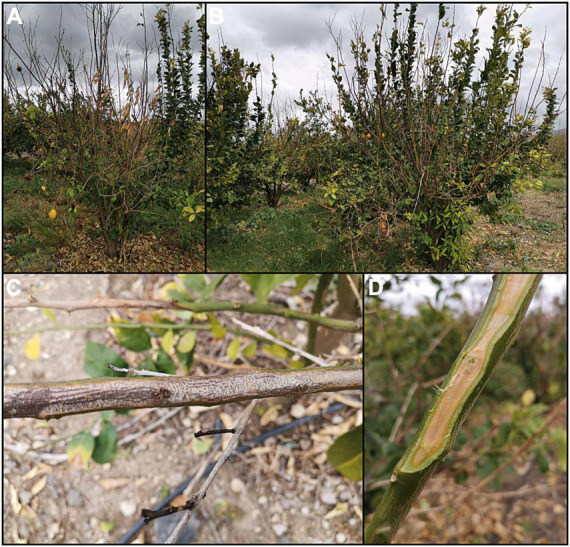
Symptoms and signs of mal secco disease (MSD) in a commercial lemon orchard (Mineo, Catania province, Italy). **(A,B)** Wilting and defoliation in young lemon trees. **(C)** Withered twig of lemon with pycnidia of *Plenodomus tracheiphilus* (pycnidia appear as scattered black spots). **(D)** Longitudinal section on a lemon twig with the typical orange-reddish discoloration of the wood.

Citrus are a major fruit crop worldwide, with a global production of about 158 million tons and an area of around 10 million hectares (FAOSTAT, 2022). Lemon is the third foremost citrus species after orange and mandarin, with an annual production of around 20 million tons of fruit and an area of 1.2 million hectares (Balmas et al., 2005). Around 48% of the global lemon production comes from countries of the Mediterranean Region and Black Sea basin. A major constraint of lemon industry in these areas is the MSD (Balmas et al., 2005; Migheli et al., 2009; European Food Safety Authority, 2014). Mal secco is a highly destructive tracheomycosis caused by the mitosporic fungus *Plenodomus tracheiphilus* (formerly *Phoma tracheiphila*), in the family Leptosphaeriaceae (Demontis et al., 2008; Figure 1). The pathogen of MSD infects the host plant by penetrating through wounds and leaf scars. The most common sanitation practice of MSD is the pruning of symptomatic branches; however, it often represents both an entry route for new infections and a source of inoculum, because of the emerging of the mycelium and spores from infected wood exposed to the air in climatic conditions favorable to the sporulation of the pathogen (Raimondo et al., 2007; Migheli et al., 2009). Consequently, it is recommended to avoid rainy or cloudy days for pruning lemon trees as these environmental conditions are conducive to infections and favor the production and dispersion of conidia of the pathogen. Another source of inoculum is represented by pycnidia, fruiting bodies differentiated on withered twigs and suckers (Raimondo et al., 2007; Migheli et al., 2009). Additionally, inoculum of the pathogen can persist for months in the soil in association with leaf and wood debris and may infect the trees through wounded roots (Migheli et al., 2009; Raimondo et al., 2010). Treatments with copper-based fungicides are commonly used to control lemon MSD, both in commercial orchards and nurseries. To be effective, multiple applications are needed (Migheli et al., 2009). However, these kinds of treatments determine concerns related to the long-lasting persistence of copper in the environment, which is then accumulated in the soil and ground-waters, with the consequent toxic effects on plants, animals and soil microbiota, and the contamination of food (Zhou et al., 2011; Tamayo et al., 2016; Abbate et al., 2019). Therefore, restrictions in the use of copper-based fungicides were introduced by the European Union (La Torre et al., 2018).

The need to minimize copper inputs in agriculture has prompted researchers to seek innovative formulations for copper-based fungicides and strategies to reduce dosages (Vicent et al., 2009). In this perspective, the possibility to include fungicidal active ingredients in highly functional gel-based formulations, could represent an effective strategy to reduce the dispersion of copper in the environment, maximizing, at the same time, its effectiveness toward MSD. The Super absorbent polymers (SAPs) are synthetic lightly crosslinked hydrophilic polymers that can absorb, swell, and retain aqueous solutions up to hundreds of times their own weight (Kabiri et al., 2003). In detail, when water interacts with the functional groups of SAP, it is drained into it by both hydrogen bonding and osmosis. Then, the water rapidly migrates into the interior of the polymer network where it is stored, determining the turning of the SAP into hydrogel. In deionized and distilled water, SAP can absorb 300 times its weight and can become a gel, but when placed in a 0.9% salt solution, the absorbency drops to 50 times its weight (Yadav and Shrestha, 2019). The total absorbency and swelling capacity are controlled by the type and grade of cross-linker used to produce the SAP. Low-density cross-linked SAPs generally have a higher absorptive capacity and swell more (Kabiri et al., 2003). These types of SAP also have a softer, stickier gel formation. High-density cross-linked polymers have a lower absorptive capacity and swell, but the gel strength is firmer and can maintain particle shape even under moderate pressure (Kabiri et al., 2003).

Super absorbent polymers have been used in agriculture for several purposes, such as to improve seed germination, save irrigation water and slowly release fertilizers (Behera and Mahanwar, 2020). However, they were not exploited to deliver fungicides for controlling plant diseases. In the present study, we demonstrated for the first time the effectiveness of a SAP saturated with a copper (II) sulfate solution in inhibiting the fungus *P. tracheiphilus*, the causal agent of MSD disease of lemon.

## Materials and methods

### *Plenodomus tracheiphilus* isolate, culture conditions, and conidial suspension preparation

The *P. tracheiphilus* isolate Pt 42 used in this study belonged to the collection of the Laboratory of Molecular Plant Pathology of the Department of Agriculture, Food and Environment, University of Catania (Catania, Italy); the species identity was confirmed in previous study (Demontis et al., 2008; GenBank accession number of the sequence of the internal transcribed spacer (ITS) region of the nuclear rRNA: AY531666).

Conidia production was stimulated by culturing the isolate on potato dextrose agar (PDA; Oxoid Ltd., Basingstoke, United Kingdom) at 25°C, in the dark, for 7 days. Then, the culture was flooded with 2 ml of sterile distilled water (SDW) and the surface of the colony was gently scraped. The suspension was collected and filtered through two layers of cheesecloth to remove mycelial fragments. The concentration of conidia suspension was finally adjusted at 10^4^ conidia/mL (a haemocytometer was used).

### *In vitro* test to assess the antifungal activity of copper (II) sulfate

The antifungal activity of copper sulfate (CuSO_4_⋅5H_2_O) against *P. tracheiphilus* isolate Pt 42 was preliminarily assayed in PDA Petri dishes by an agar diffusion test (Stracquadanio et al., 2020; El boumlasy et al., 2021). The copper sulfate, in aqueous solution, was tested at the following concentrations: 0.0, 15.0, 50.0, 100.0, and 200.0 mg/mL, corresponding about to a concentration of Cu^2+^ ions of 0.0, 60.0, 200.0, 400.0, and 801.0 mM, respectively. For the test, 500 μl of the conidial suspension (10^4^ conidia/mL) of the pathogen were homogeneously spread over the entire surface of a PDA Petri dish; then, five wells (diameter 5 mm) were made by using a cork borer and filled with 60 μl of each copper sulfate solution. The dishes were sealed with Parafilm^®^ and incubated for 3 days at 25°C, in the dark. The test was performed in triplicate and was repeated twice; the results were evaluated by measuring the diameter of the inhibition halos of the pathogen.

### Determination of minimum inhibitory concentration and minimum fungicidal concentration of copper (II) sulfate

The minimum inhibitory concentration (MIC), defined as the lowest concentration of the treatment that inhibits the visible fungal growth, was determined by a microdilution method. The determination of the MIC value was carried out according with the method of Kubra et al. (2013) with slight modifications. The test was performed in 2 ml tubes by incorporating 400 μL of autoclave-sterilized aqueous solutions of copper (II) sulfate at various concentrations (6.242, 12.484, 18.726, 24.968, 31.210, 37.452, 43.694, 49.936, 56.178, and 62.420 mg/mL) into 400 μL of sterile potato dextrose broth (PDB). Then, 200 μL of conidial suspension (10^4^ conidia/mL) were added to each tube and incubated at 25°C for 7 days, in the dark. A mixture of sterile PDB and the conidial suspension was used as a control. The final concentrations Cu^2+^ ions in test tubes were then 10.0, 20.0, 30.0, 40.0, 50.0, 60.0, 70.0, 80.0, 90.0, and 100.0 mM. After the incubation period, the MIC was determined as the concentration of copper (II) sulfate which determined no visible cloudiness in the tubes, indicating that no pathogen growth occurred.

The minimum fungicidal concentration (MFC) was determined by transferring 10 μL from each tube where cloudiness was observed, onto PDA Petri dishes. The inoculated dishes were incubated at 25°C for 3 days, in the dark. The MFC was finally evaluated as the plated concentration that did not lead to any mycelial growth after the incubation period. Both tests (MIC and MFC) were performed in triplicate and repeated twice.

### Super absorbent polymer

The Super absorbent polymer (SAP) employed in this study was the STOCKOSORB^®^ 660 (CREASORB, Krefeld, Germany), a polyacrylic acid–potassium salt, crosslinked based hydrogel specifically formulated for water and nutrient retention in soil and potting mixtures in agriculture.

### Determination of the absorption capacity of super absorbent polymer

Five aqueous solutions of copper sulfate (II) were prepared at the concentrations 0.0, 32.0, 10.0, 20.0, and 59.0 mg/mL, corresponding about to 0, 8, 39, 79, and 236 mM of Cu^2+^ ions, respectively. For all concentrations, the absorbency was determined by immersing 100 mg of SAP in a constant volume of aqueous solution. Then, all mixtures were stirred at 150 rpm at room temperature until the SAP reached the equilibrium swelling. The SAP particles with entrapped Cu^2+^ [hereinafter “super absorbent polymer containing copper (SAP-Cu)”] were collected by filtration and the concentration of copper ions was determined by atomic absorption spectrometer (AAS) in remaining solutions. For any tested concentration, results are proposed as mean of three technical replicates.

### Evaluation of copper ions release kinetics of super absorbent polymer containing copper

In order to study the attitude of the SAP-Cu to release copper ions, the kinetics of Cu^2+^ release was simulated on a laboratory scale by placing the SAP-Cu in contact with water-soaked cellulose.

Different concentrations of SAP-Cu were tested. Each SAP-Cu hydrogel was prepared in 100 mL beakers by immersing 500 mg of SAP in 10 ml of copper (II) sulfate solutions at different concentrations (8, 39, 79, and 236 mM of Cu^2+^ ions) under stirring at 150 rpm for 10 min. Once the SAP reached the equilibrium swelling and the active ingredient (Cu^2+^) was loaded into the polymeric material, each SAP-Cu hydrogel was put in contact with cellulose pads that were presoaked with 5 ml of water. The cellulose pads with SAP-Cu in contact on the top were placed inside Petri dishes, sealed with Parafilm^®^ (in order to prevent water evaporation during storage) and incubated until specific time-points (24, 48, 72, and 96 h after the beginning of SAP-Cu/water-soaked cellulose contact). In order to extract the Cu^2+^ ions transferred from the SAP-Cu hydrogel to the cellulose pad, at each time-point each cellulose pad was collected and submerged in 50 ml of hot water for 10 min; finally, the concentration of Cu^2+^ ions in the solutions was determined by UV-VIS spectroscopy (Spectroquant^®^ by Supelco analytic kit and the UV-VIS spectrometer Jasco V-670 were used).

For each concentration of SAP-Cu hydrogel, the values of concentration of Cu^2+^ ions released in the cellulose were expressed versus the time and represented as best fit calculated by the equation:


(1) $$C=C\,m\,a\,x-\left(C\,m\,a\,x-C\,o\right)\,e^{-K\,t}$$

where *Co* is the initial concentration of Cu^2+^ in the cellulose, *Cmax* represents the maximum concentration of Cu^2+^ that can be released after a time so long as to be considered infinite (plateau region), *t* is the time and *K* is the constant of release of Cu^2+^ ions of the SAP-Cu hydrogel.

The dependence of the maximum releasable concentration of Cu^2+^ by SAP-Cu on the concentration of the initially adsorbed copper ions solution was also described.

### Evaluation of the attitude of super absorbent polymer containing copper to fully release all the adsorbed Cu^2+^ ions

The attitude of SAP-Cu to fully release all the adsorbed Cu^2+^ ions to another material until the reaching of the equilibrium was additionally checked by a specifically accomplished test. To this aim, 500 mg of SAP containing 10 ml of copper solution at the concentration of 236 mM of Cu^2+^ ions were placed in contact with a cellulose pad presoaked with 5 ml of water and incubated into parafilm sealed Petri dishes for 7 days. At specific time-points (1, 2, 3, 4, 5, 6, and 7 days from the beginning of SAP-Cu/water-soaked cellulose contact), the cellulose pad was collected and substituted with a new one until the last time point (7 days). At each time-point, the amount of Cu^2+^ ions transferred from SAP-Cu hydrogel to the cellulose pad was determined by the same extraction procedure and UV-VIS spectroscopy methodology outlined in 2.5.

### Effect of super absorbent polymer containing copper on fungal mycelial growth

To assess the antifungal activity of SAP-Cu on the mycelial growth of *P. tracheiphilus* isolate Pt 42 the following procedure was applied: SAP-Cu and “SAP-sterilized ionized water (SAP-H_2_O) (control 1)” were prepared by mixing one gram of SAP with 20 ml of copper solution at the concentration of 236 mM of Cu^2+^ ions or with sterilized ionized water as a control, respectively. The SAP-Cu and the SAP-H_2_O were stirred for 10 min at room temperature and autoclaved for 20 min at 121°C. Then, the top layer of PDA plates was covered with the SAP-Cu or SAP-H_2_O. Petri dishes were sealed with Parafilm^®^ and stored at room temperature. After 24 h of incubation, the SAP-Cu and SAP-H_2_O were removed with a sterilized spatula.

The PDA plates preliminarily treated (as reported above) with SAP-Cu or SAP-H_2_O, and an additional group of untreated PDA control plates (control 2) were inoculated with two agar plugs (5 mm diameter, each) cut from the edge of the actively growing colony of *P. tracheiphilus* isolate Pt 42. All the plates were sealed with Parafilm^®^ and incubated at 25°C for 10 days. The antifungal effect of SAP-Cu and SAP-H_2_O on mycelial growth was expressed as percent inhibition of mycelial growth (%) and calculated according with the following formula (Farzaneh et al., 2015):


(2) $$\text{Inhibition}\%=\frac{\text{C–T}}{\text{C}}\times 100$$

Where C is the mean colony diameter (mm) of “control 2” colonies, and T is the mean colony diameter (mm) of the SAP-Cu or SAP-H_2_O treated group. Each treatment included three replicates. The experiment was repeated twice.

### Evaluation of super absorbent polymer containing copper effectiveness in affecting *Plenodomus tracheiphilus* viability in naturally infected twigs

**FIGURE 2:**
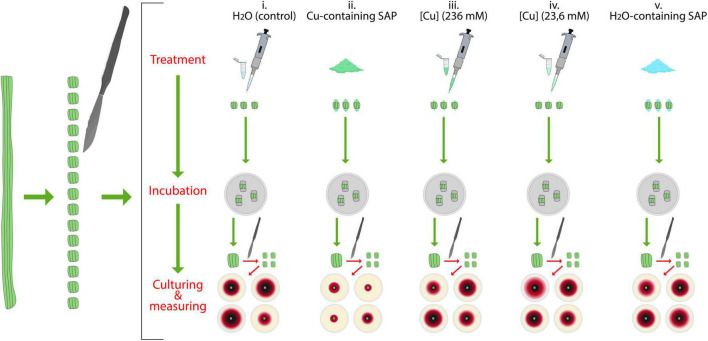
Schematic representation of the test for the evaluation of the capacity of super absorbent polymer containing copper (SAP-Cu) to inhibit *Plenodomus tracheiphilus* growth in naturally infected twigs.

The test was carried out by using *P. tracheiphilus*-naturally MSD infected twigs (1 cm diameter) collected from symptomatic lemon trees in a local citrus orchard (Mineo, Catania province, Italy); all tests were conducted in triplicate, by processing in the same way three distinct symptomatic twigs. The experimental scheme of this experiment is reported in Figure 2. For each twig, 15 pieces (length = 1 cm, each) were obtained by cutting with a sterilized scalpel; then, all the pieces were disinfected by dipping in NaClO (3%) for 3 min and subsequent rinsing in SDW for 1 min. Disinfected twig segments were randomly organized in groups of three. Then, both edges of any segment belonging to each group of three was treated with one of the following substances: (i) sterilized distilled water

(SDW, control)–50 μl per edge; (ii) SAP-Cu (236 mM of Cu^2+^ ions)–100 mg per edge; (iii) CuSO_4_⋅5H_2_O-aqueous solution (236 mM of Cu^2+^ ions)–50 μl per edge; (iv) CuSO_4_⋅5H_2_O-aqueous solution (23.6 mM of Cu^2+^ ions)–190.67 μl per edge (this is an amount chosen on the basis of the absorption capacity of SAP and the kinetics of release of Cu^2+^ ions by 100 mg of SAP-Cu at 236 mM of Cu^2+^ ions–as used in treatment, i.e.; see results in sections “Absorption capacity of super absorbent polymer” and “Kinetics of release of copper of Cu-containing SAP” paragraphs); (v) SAP-H_2_O (100 mg per edge). Both edges of each twig segment were then sealed with Parafilm^®^ and then wrapped with aluminum foils (3 cm × 3 cm pieces). Twig segments from all treatments were finally incubated in a humid chamber (Petri dish) for 72 h at 25°C and 80% of relative humidity.

In order to determine the influence of any treatment on the viability of *P. tracheiphilus*, after the incubation period, each 1-cm-long segment was reduced in 5 mm × 5 mm sub-pieces that were singularly plated onto streptomycin sulfate (Sigma-Aldrich, St. Louis, MO, United States)-amended (0.250 g/L) PDA plates. Plates from each treatment were then incubated at 25°C for 72 h, in the dark. After the incubation period, the diameter of *P. tracheiphilus* growing colonies was recorded [the specific identity of *P. tracheiphilus* growing colonies was confirmed by q-RT PCR performed in accordance with Demontis et al. (2008)].

The influence of any treatment in affecting the viability of *P. tracheiphilus* was then expressed in terms of mean colony diameter.

### Evaluation of copper ions exchange with the vascular system of the plant

The spatial distribution and concentration of Cu^2+^ ions in SAP-Cu treated branch of affected tress was established by time-of-flight secondary ion mass spectrometry (ToF-SIMS) in 10 mm × 10 mm twig segments placed in contact for 24 h with SAP-Cu at 236 mM of Cu^2+^ ions; twigs immersed in Cu containing solution (236 mM) for 2 min were used as comparative control. Results are proposed as chemical high-resolution, static ToF-SIMS images acquired with a ToF-SIMS IV (ION-ToF) instrument using a Bi^3+^ analysis beam (bunched mode, 25 keV, ∼0.1 pA, 256 × 256 pixels, single scan, rastered over 500 μm × 500 μm). The primary ion fluence was kept below 1 × 10^12^ ions × cm^–2^ (Tuccitto et al., 2018). Because the area to be analyzed exceeded the maximum raster size of the primary ion beam, the acquisition of a chemical image of both sets of twigs required the macroraster mode feature of the instrument to be exploited, collecting several stacked single raster scans; thus, 5 mm^2^ × 5 mm^2^ areas were investigated.

## Results

### *In vitro* preliminary tests, minimum inhibitory concentration, and minimum fungicidal concentration

**FIGURE 3:**
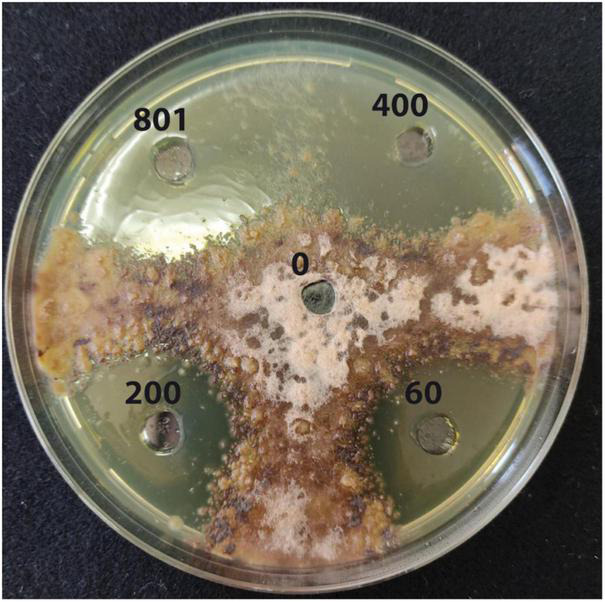
Agar diffusion test. Inhibition halos of mycelium growth of *Plenodomus tracheiphilus* induced by copper sulfate solution at different Cu^2+^ concentrations (0, 60.0, 200.0, 400.0, and 801.0 mM), after 3 days of incubation at 25°C.

Results from the *in vitro* test showed a significant inhibitory effect of copper (II) sulfate toward the mycelial growth of *P. tracheiphilus* isolate Pt 42 at all concentrations tested (Table 1 and Figure 3). The highest dose (Cu^2+^ at 801.0 mM) determined the most marked inhibitory effect on pathogen growth, with a mean diameter of the inhibition halo of 49.27 ± 0.55 mm, while the lowest dose (Cu^2+^ at 60.0 mM) determined a mean inhibition halo of 12.47 ± 0.45 mm. The MIC and the MFC of copper (II) sulfate toward of *P. tracheiphilus* isolate Pt 42 were 37.452 and 43.694 mg/mL, corresponding to a concentration of Cu^2+^ of 60 and 70 mM, respectively (Table 2).

**TABLE 1:** Inhibitory effects of copper sulfate solution at different Cu^2+^ concentrations (0.0, 60.0, 200.0, 400.0, and 801.0 mM) on the mycelium growth of *Plenodomus tracheiphilus* Pt 42, as determined with the agar diffusion test by measuring the diameter of the inhibition halo around the wells, after 3 days of incubation at 25°C.

Cu^2+^ (mM)	Diameter of the inhibition halo (mm) (mean ± standard deviation)
0.0	0 ± 0 a
60.0	12.47 ± 0.45 b
200.0	30.77 ± 0.30 c
400.0	38.23 ± 0.25 d
801.0	49.27 ± 0.55 e

Values sharing different letters are statistically different according with Tukey’s honestly significant difference (HSD) test (*P* ≤ 0.05).

**TABLE 2:** Minimum inhibitory concentration (MIC) and minimum fungicidal concentration (MFC) determined by Cu^2+^ in *Plenodomus tracheiphilus*.

	Cu^2+^ (mM)	
Isolate code	MIC	MFC
Pt 42	60.0	70.0

### Absorption capacity of super absorbent polymer

**FIGURE 4:**
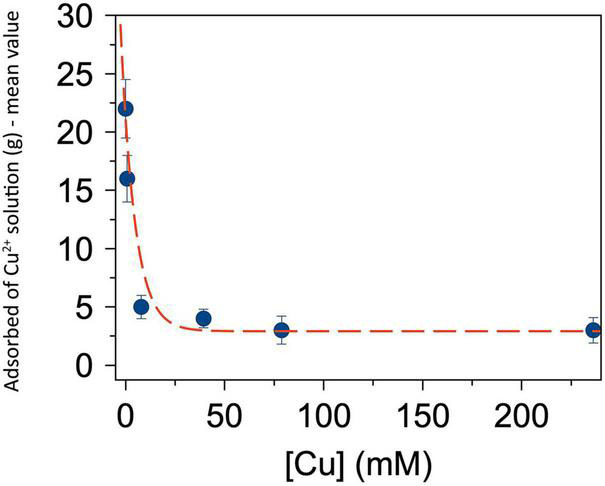
Mean amount (g) of Cu^2+^ solution adsorbed by 100 mg super absorbent polymer (SAP) on the respective value of Cu^2+^ concentration (mM) in the same solution used for producing the super absorbent polymer containing copper (SAP-Cu). The red dotted line helps the reader’s eye. Bars represents standard deviation (SD).

The results of the absorption test carried out by employing different concentrations of copper (II) sulfate solution are shown in Figure 4. The absorption values were inversely proportional to the concentration of copper (II) sulfate. The highest quantity of absorption was obtained with ionized water, which increased the SAP weight of ca. 200 times its original weight. Conversely, the lower quantity of absorption was obtained with copper (II) sulfate solutions at 236 mM which increased the original SAP weight of ca. 30 times.

### Kinetics of release of copper of Cu-containing super absorbent polymer

**FIGURE 5:**
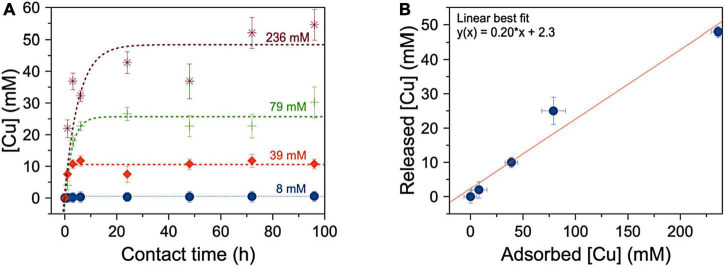
**(A)** Mean amount of copper released from the hydrogel to the soaked cellulose pad as a function of the time of contact (expressed in hours–h); dotted lines describe the linear fits of the kinetics of Cu^2+^ ions release. **(B)** Dependence of the maximum amount of Cu released by super absorbent polymer (SAP) after 24 h (*Cmax* calculated by the fit reported in panel **A)** on the concentration of the copper ions solution adsorbed by the SAP to produce the hydrogel. Bars represents SD.

The kinetics of copper ion release of SAP-Cu hydrogel is described by the curves represented in Figure 5A. In detail, the fitting parameters show that, for SAP-Cu hydrogel at all the tested concentrations, the maximum concentration of Cu^2+^ that can be released (*Cmax*, the plateau region of each curve) is reached after 24 h of incubation on the top of the cellulose pads.

Data reported in Figure 5B show the dependence of the concentration of released copper by SAP on the concentration of the solution used to form the hydrogel (namely, copper absorbed by the SAP for the preparation of SAP-Cu). The value of the angular coefficient of the line which best fits the data of concentration of released vs. adsorbed Cu^2+^ by the SAP, indicates that the hydrogel is able to release ca. the 20% of the contained copper.

### Attitude of super absorbent polymer containing copper to fully release all adsorbed Cu^2+^ ions

**FIGURE 6:**
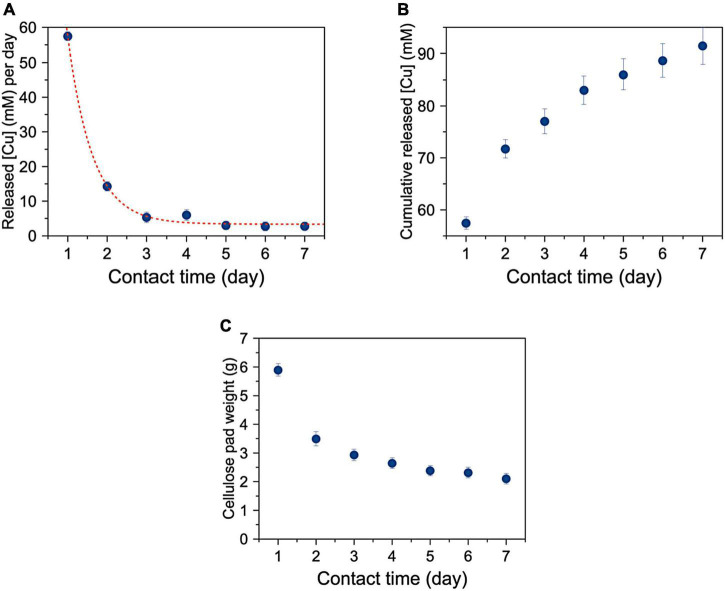
**(A)** Mean concentration of released Cu per day as a function of time with a cellulose pad renewed each 24 h of contact. Red line is a first-order exponential best fitting experimental points; **(B)** Cumulative Cu released as a function of time; **(C)** Mean weight of cellulose pads used for 7 days experiment and renewed after of 24 h of contact.

Figure 6A shows that the amount of copper released per day by SAP-Cu decreases according with a first-order exponential trend. Figure 6B shows the cumulative amount of copper released in 1 week by changing the cellulose pad every 24 h. Figure 6C shows that the weight of the water-soaked cellulose pad from decreased from about 6 g (5 ml water and 1 g cellulose) to about 2 g.

### *In vitro* effects of super absorbent polymer containing copper on mycelial growth and conidial survival

**FIGURE 7:**
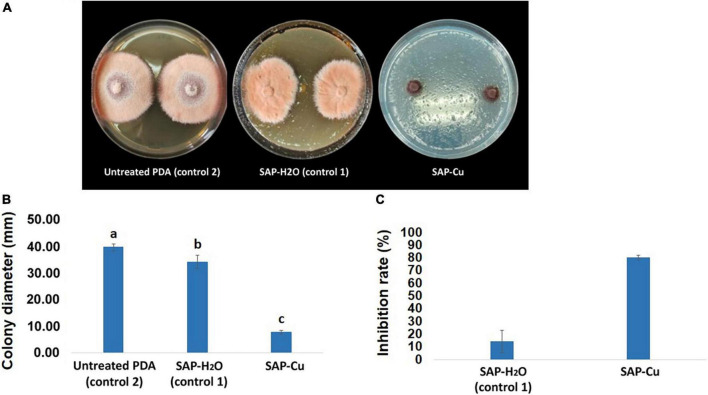
Inhibition effects of super absorbent polymer (SAP) containing copper at the concentration of 0 (SAP-H_2_O–control 1) and 236 mM super absorbent polymer containing copper (SAP-Cu) over untreated potato dextrose agar (PDA) (control 2) on the mycelial growth of *Plenodomus tracheiphilus* isolate Pt 42 after 10 days of incubation at 25°C on PDA. **(A)** Comparison of cultures from different treatments, **(B)** mean colony diameter (mm), and **(C)** mean inhibition rate (%). In B, columns with different letters are significantly different according with the Tukey’s HSD (Honestly Significant Difference) *p* = 0.05. Bars represents SD.

Results of the mycelium growth test evidenced that the SAP-Cu at the concentration of 236 mM exerted a macroscopically evident inhibitory effect on *P. tracheiphilus* isolate Pt 42 growth (Figure 7A). Super absorbent polymer containing copper treatment on mycelial growth of colonies was significantly different from colonies treated with SAP-H_2_O or grown only on PDA (control). After 10 days of incubation, the mean colony diameter of the *P. tracheiphilus* Pt 42 was about 39.83 mm in untreated PDA control plates. The mean diameter of colonies grown on SAP-H_2_O and SAP-Cu was 34.33 mm in and 7.83 mm, respectively (Figure 7B); these values corresponded to a mycelial growth inhibition of 14 and 80%, respectively (Figure 7C).

### Evaluation of super absorbent polymer containing copper effectiveness in affecting *Plenodomus tracheiphilus* viability in naturally infected twigs

**FIGURE 8:**
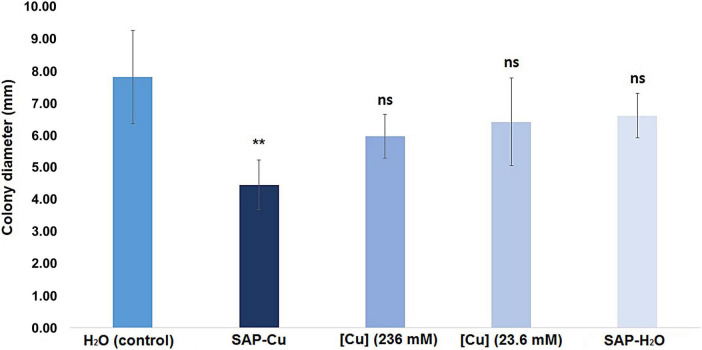
Effect of Cu-containing SAP (SAP-Cu), CuSO_4_⋅5H_2_O-aqueous solution, both at the concentration of 236 mM of Cu^2+^, CuSO_4_⋅5H_2_O-aqueous solution at the concentration of 23.6 mM of Cu^2+^ and H_2_O-containing SAP (SAP-H_2_O) on the mycelial growth of *Plenodomus tracheiphilus* in naturally infected twigs (cfr. Figure 2). Error bars represent the standard deviation of the means (*n* = 36). Columns with asterisks are statistically different according with Dunnett’s test (**P < 0.01, ns = not significant), compared to the control.

Results from this test revealed that the diameter of colonies of *P. tracheiphilus* recovered from twig cuttings, collected from naturally infected lemon trees, treated with SAP-Cu (236 mM of Cu^2+^ ions) (treatment ii.) was significantly smaller than the diameter of colonies from twig cuttings treated with distilled water (control; treatment i.). Conversely, none of the other treatments (ii., iii., and iv.) differed significantly from the control twigs (Figure 8).

### Evaluation of copper ion distribution in lemon twigs treated with super absorbent polymer containing copper

**FIGURE 9:**
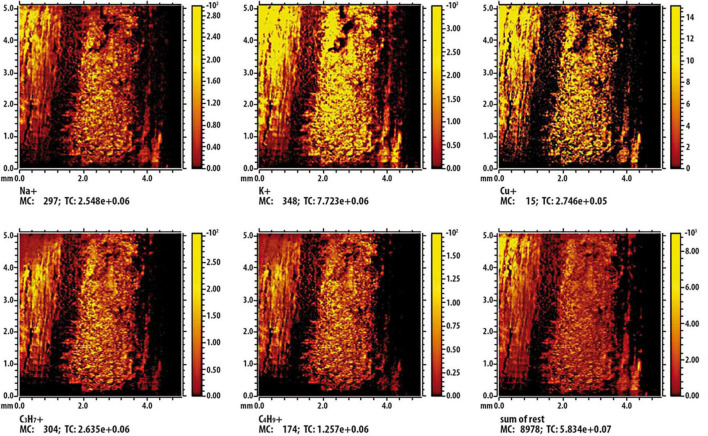
Time-of-flight secondary ion mass spectrometry (ToF-SIMS) false-color image of some characteristic ions on the surface of the twig section immersed in a copper solution. The inset labeled “sum of rest” represents the sum of the other signals present in the spectrum but not shown in the figure.

**FIGURE 10:**
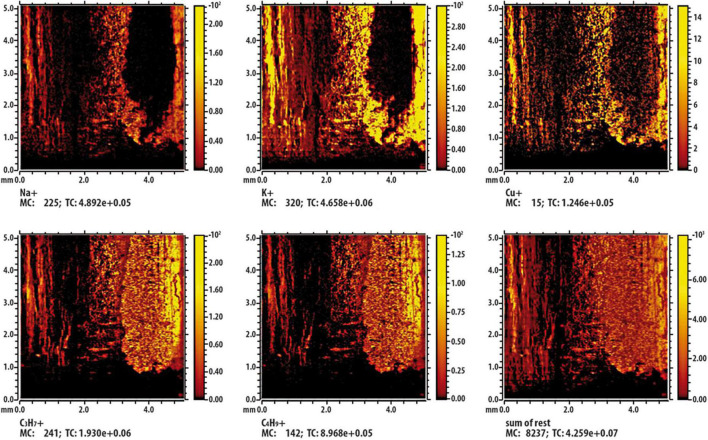
Time-of-flight secondary ion mass spectrometry (ToF-SIMS) false-color image of some characteristic ions on the surface of the twig section being in contact with Cu containing super absorbent polymer (SAP) containing copper (SAP-Cu). The inset labeled “sum of rest” represents the sum of the other signals present in the spectrum but not shown in the figure.

Results from ToF-SIMS analysis are showed as false-color images (Figures 9, 10); in detail, shining colors indicate high signal intensity for the detected ion; conversely, dark colors indicate low (or no) signal intensity for that ion. In Figure 9, which report results of the control twig [namely, a twig immersed in Cu containing solution (236 mM) for a 2 min], the mapping of intensity of each analyzed ions indicates that, being all the images very similar and overlapping, the different observed intensities of signal can be exclusively attributable to the effect of the surface roughness of the twig. Figure 10 shows the results of the investigation carried out on a twig that was placed in contact for 24 h with SAP-Cu at 236 mM of Cu^2+^ ions. In this case, not all images appear superimposable. In particular, the signals related to Na^+^, K^+^, and Cu^+^ ions (contained in the SAP) are very similar to each other and are complementary (albeit partially) to the signals related to organic wood fragments. In addition, the images show a distribution of the detected ions that is not uniform, but rather oriented along certain preferential paths aligned with the main axis of the twig.

## Discussion

Super-absorbent polymers are used in agriculture thanks to their capacity to store/release high amounts of water solution, determining consequently significant improvements of several activities related to cultivations, such as seed germination, save water for irrigation, and increase crops and fruit yields; they are also used to slowly release fertilizer, improving the efficiency of the applications (Behera and Mahanwar, 2020). Copper-containing compounds have been used as pesticides since the end of the XIX century. Their effectiveness has led to an extensive use to control plant pathogens worldwide, especially in organic farming, where synthetic pesticides are banned. However, as a consequence of growing concerns about the long-lasting environmental sustainability of copper-containing pesticides, the employment of these chemicals in agriculture has been increasingly restricted. The research is consequently driven toward ecologically safe strategies for controlling plant diseases; these include the use of antagonists of pathogens (Tucci et al., 2011; Pérez-Tornero et al., 2012; Kalai-Grami et al., 2014, 2016; La Spada et al., 2020; Hammami et al., 2022), bioproducts (Pangallo et al., 2017a,b, 2021; Belgacem et al., 2019; Stracquadanio et al., 2020, 2021; El boumlasy et al., 2021), enhancer of natural plant defense mechanisms (La Spada et al., 2021), as well as safe chemicals (Fallanaj et al., 2016). Unfortunately, the management of some plant diseases is complicated by aspects related with the same “nature” of the pathogen, determining the partial or total ineffectiveness of the majority of available means of control. This is the case of lemon mal secco disease, where the restricted host-range of the pathogen together with its remarkable virulence, its vascular localization and the long-lasting resistance structures (Migheli et al., 2009), reduces the range of choices of effective control strategies to pruning alone followed by copper sanitization of cuttings (Migheli et al., 2009; Nigro et al., 2011). In this perspective, the development of formulations that reduce the dispersion of copper in the environment and, at the same time, control the disease, is a must. Several studies have already investigated the effectiveness of copper-based formulations suitable for a controlled delivery of Cu^2+^ ions. A Cu^2+^ cross linked alginate hydrogel resulted markedly effective against both gram positive and negative bacteria (Klinkajon and Supaphol, 2014). Additionally, the Cu^2+^ incorporated into carboxymethyl chitosan (CMCh) supramolecular hydrogels showed marked antibacterial properties against *Staphylococcus aureus* and *Escherichia coli* (Wahid et al., 2017). Moreover, Cu^2+^ released from a montmorillonite biocomposite exhibited a high antifungal activity against *Pycnoporus cinnabarinus*, *Pleurotus ostreatus* and *Candida albicans* (Malachová et al., 2011; Jiao et al., 2017). Furthermore, Cu^2+^ released from copper nanoparticles showed strong growth inhibitory effects against several fungal plant pathogens, including *Fusarium oxysporum*, *Fusarium solani, Fusarium culmorum*, *Fusarium equiseti*, *Aspergillus niger*, *Aspergillus flavus*, *C. albicans* and *Neofusicoccum* spp. (Bramhanwade et al., 2016; Rai et al., 2018; Ntasiou et al., 2021).

The present study is the first pioneering application of a SAP in plant disease control. Because of its physical properties, a SAP perfectly meets the requirements of reducing the environmental dispersion of the Cu^2+^ by acting as a reservoir for the controlled release of Cu^2+^ ions while preventing, at the same time, excessive soil and groundwater contamination resulting from the leaching of the copper ion. In the present study, the potential of a SAP saturated with a copper (II) sulfate solution in controlling MSD in lemon trees was investigated. To this purpose, a preliminary investigation has been carried out to quantify the antifungal proprieties of copper (II) sulfate against *P. tracheiphilus* by determining MIC and MFC. The absorption capacity of the SAP and the associated kinetics of the release of copper ions from the SAP-Cu were evaluated. Hence, SAP-Cu was screened for its effectiveness in inhibiting *in vitro* the fungal mycelial growth of *P. tracheiphilus*. Finally, antifungal tests were carried out to evaluate both the effect of the SAP-Cu in the control of MSD in naturally infected lemon twigs and the distribution and concentration of Cu^2+^ ions in lemon branches treated with the newly developed SAP-Cu.

Results from the agar diffusion test together with those from MIC and MFC provided data for the evaluation of the most effective concentrations of copper (II) sulfate in inhibiting the growth of *P. tracheiphilus*. The antimicrobial activity of copper (II) sulfate is widely reported in scientific literature (Cady et al., 2011; Iakovidis et al., 2011; Bouson et al., 2017; Sinisi et al., 2019). Several studies highlighted that copper (II) sulfate is a plant protectant with a broad spectrum of activity toward a broad range of diseases caused by both fungi and bacteria (Al-Holy et al., 2010; Judet-Correia et al., 2011; dos Santos et al., 2019). Its effect is due to the release of free Cu^2+^ ions, which, in turn, can generate reactive hydroxyl radicals that may damage lipids, DNA, proteins, as well as any membrane with which they interact (Rai et al., 2018; Malhotra et al., 2020).

The hydrogel used in the present study, a SAP based on cross linked polyacrylic acid co-potassium salt, is insoluble in water and organic solutions, and it swells to a gel upon contact with aqueous solutions. Hydrogels possess an enormous capacity in retaining water and absorbing heavy metal ions from water. This capacity depends on their unique three-dimensional structure, constituted by networks of cross-linked polymers, and their chemical responsive functional groups (Mohan et al., 2005; Puglisi et al., 2012; Cacciola et al., 2015; Muya et al., 2016; Shalla et al., 2019; Sun et al., 2019; Chowdhury et al., 2021; Perumal et al., 2021).

The results of the absorption test highlighted that the absorption capacity of SAP was inversely proportional to the concentration of Cu^2+^ ion. In detail, the highest absorption was obtained with ionized water, which increased the weight of the dry SAP of ca. 200 times; this is consistent with previous reports showing that superabsorbent polymers can absorb an amount of water at least up to 100 times the volume of the original dry polymer (Liu and Guo, 2001; Park and Kim, 2001; Sohn and Kim, 2003; Hansen et al., 2005). Conversely, the lowest absorption was obtained with the copper (II) sulfate aqueous solution at 236 mM of Cu^2+^ ions, which increased the dry weight of the polymer of ca. 30 times. This result can be explained assuming that the increase of the concentration of the ion Cu^2+^ decreases the absorptive capacity of SAP. It could depend on the saturation of hydrogel sites that were completely filled with ions and consequently no vacant sites were available for entrapping more ions and water. These hypotheses are in agreement with other studies which revealed that the metal absorption is, generally, limited by metal diffusion inside the hydrogel and the hydrogel-water interfacial area (Katime and Rodríguez, 2001; Kostić et al., 2007). Therefore, if the amount of copper loaded into the hydrogel has to be increased, a carefully analysis should be preliminarily performed because a higher concentration of copper in the solution could reduce the amount of adsorbed ions.

With reference to the kinetics of copper ion release, results from this study highlighted that the amount of copper released from the hydrogel never reached the total amount of Cu^2+^ ions contained (nominally 236 mM), but a part remained unavailable for delivery even after prolonged contact. It must also be stressed that, as the concentration of copper in the hydrogel decreased, there was a greater capacity for the SAP to adsorb water from the cellulose pad with which it was in contact. This effect can be explained considering that a lower concentration of copper in the SAP makes the carboxyl groups responsible for water loading more available by forming hydrogen bonds with water molecules; therefore, the lower the copper, the higher the amount of water sucked up by the SAP. This interpretation was also supported by the observation of a progressive decreasing of weight of the water-soaked cellulose pad, which indicated the release of ion Cu^2+^ from the hydrogel and water intake from the cellulose to the hydrogel once the Cu^2+^ decreases in the polymeric network.

Results from the *in vitro* preliminary tests, together with those from SAP absorption capacity and the kinetics of copper ion release, provided elements for the selection of the most suitable and performing Cu-containing SAP (both in terms of absorption capacity and release of adsorbed Cu) to be tested *in vitro* as well as in cuttings from lemon twigs naturally infected by *P. tracheiphilus*. Therefore, the choice was on the SAP at 236 mM of Cu^2+^ ions, whose maximum concentration of released Cu^2+^ ions was about 49.5 mM; although this concentration was about the 20 and 30% lower than MIC and MFC, respectively, it was the best compromise to achieve the right balance between maximum uptake and maximum release of Cu^2+^ ions.

Results from the *in vitro* test carried out by using the SAP-Cu reported a marked inhibition of the mycelial growth of the pathogen, indicating that, although the SAP-Cu hydrogel released an amount of Cu^2+^ ions corresponding to a concentration lower than measured MIC and MFC, the copper ions were properly delivered into the PDA medium. Additionally, in the *in vitro* test, a slight growth inhibition was also observed in cultures grown in PDA treated with SAP-H_2_O. This result could be interpreted by considering the absorption/release properties of the SAP hydrogel employed in this study. The SAP might have adsorbed nutrients from the culture medium and releasing at the same time water, which, in turn, might have been adsorbed by the PDA, resulting in the dilution of the remaining nutrients. Further research is underway to confirm this hypothesis.

The performance of SAP-Cu was finally tested in naturally infected twigs. Two aspects were evaluated: (i) the efficacy of SAP-Cu in reducing the viability of *P. tracheiphilus*; (ii) the spatial distribution and concentration of Cu^2+^ ions resulting from the treatment of lemon twigs with SAP-Cu.

Overall, results evidenced that the cultures of *P. tracheiphilus* grown from SAP-Cu treated lemon twigs had a diameter significantly lower than those of cultures recovered from all the other treatments. Interestingly, none of the treatments with Cu^2+^ ions in solution (e.g., aqueous solution of Cu^2+^ ions at 236 and 23.6 mM) significantly inhibited the growth of the pathogen compared with the control.

Also considering that from twigs treated with SAP-H_2_O, *P. tracheiphilus* cultures were not inhibited compared with the control, the inhibition in the growth of cultures developed from SAP-Cu treated lemon twigs can be attributed to the action of Cu^2+^ ions. Therefore, it can be inferred that the application of Cu^2+^ ions to lemon twigs by SAP-Cu significantly affects the viability *P. tracheiphilus* and is more efficient than the treatment with aqueous solutions containing Cu^2+^.

A possible interpretation of this result can be provided by considering the results from ToF-SIMS analysis, which described the spatial distribution of copper ions in lemon twigs treated with SAP-Cu and aqueous Cu^2+^solution. Overall, the results showed that both treatments resulted in the adsorption of Cu^2+^ ions; however, by comparing the chemical images of the two treatments, some aspects can be highlighted. Whenever the twigs adsorb Cu^2+^ ions from the aqueous copper solution, a uniform distribution of Cu^2+^ ions in the plant segment is observed. If Cu^2+^ ions are transferred to the twig from the SAP-Cu hydrogel, they are not uniformly distributed in the plant segment, but are preferentially located along the microfluidic channels, particularly along the xylem vessels. This can be explained by taking into account that the SAP-Cu hydrogel exchanges water (including ions) with other water-containing materials, which, in this case, are the xylem vessels of the twigs.

Therefore, as *P. tracheiphilus* progressively moves forward in the plant from the site of infection through the xylem vessels, it can be hypothesized that the results of the test carried out in this study in naturally infected lemon cuttings are a direct consequence of the improved efficacy of SAP-Cu, which enhanced the delivery of Cu^2+^ ions in the tissues preferentially colonized by *P. tracheiphilus*, thus affecting its viability to a greater extent than Cu^2+^ ions applied to the twigs by aqueous solutions containing Cu^2+^. Localization of Cu^2+^ ions within the xylem vessels could result in an additional positive effect in controlling mal secco infections in lemon plants. In fact, the progressive movement of *P. tracheiphilus* within the infected tissues is typically supported by phialoconidia, propagules of the pathogen that move into the xylem by taking advantage of the plant transpiration flow (Migheli et al., 2009); therefore, the presence of Cu^2+^ ions in the xylem vessels could also affect the viability of these propagules, leading to their inactivation.

## Conclusion

In summary, this study shows that SAP (STOCKOSORB^®^ 660) is an efficient carrier of Cu^2+^ ions with antimicrobial properties and paves the way to new strategies in controlling fungal plant diseases. *Plenodomus tracheiphilus*, the causal agent of MSD, a destructive tracheomycosis of lemon, was the case study to investigate the potentialities of SAP containing a fungicidal active ingredient. The capacity of this hydrogel to absorb Cu^2+^ ions and regulate the flux of liquids through which Cu^2+^ ions are delivered inside the plant vascular system, confers to this innovative Cu-containing formulation a potential protection and localized eradication activity against other pathogens, such as fungi causing wood rot and cankers of citrus, fruit trees and grapevine (Roccotelli et al., 2014; Aloi et al., 2021; Moretti et al., 2021; Bolboli et al., 2022). In addition, the ability of SAP to slowly release Cu^2+^ ions could be exploited in the control of soil borne plant diseases in soilless culture as this formulation would offer the advantage of preventing the leaching of this heavy metal and groundwater contamination.

## Data availability statement

The raw data supporting the conclusions of this article will be made available by the authors, without undue reservation.

## Author contributions

SOC, NT, AP, and AD: conceptualization. SOC, NT, SEb, and FLS: methodology. FLS, SEb, and NT: software. SOC, NT, AL, AP, and AD: validation and writing—review and editing. SEb, FLS, and NT: formal analysis and investigation, data curation, and writing—original draft preparation. SOC, AP, NT, and AL: resources. SOC: visualization. AD and SOC: supervision. All authors have read and agreed to the published version of the manuscript and approved the submitted version.
